# *N*-(2,5-Dimethyl­phen­yl)succinamic acid monohydrate

**DOI:** 10.1107/S1600536811024937

**Published:** 2011-07-02

**Authors:** B. S. Saraswathi, Sabine Foro, B. Thimme Gowda

**Affiliations:** a Department of Chemistry, Mangalore University, Mangalagangotri 574 199, Mangalore, India; b Institute of Materials Science, Darmstadt University of Technology, Petersenstrasse 23, D-64287 Darmstadt, Germany

## Abstract

In the title compound, C_12_H_15_NO_3_·H_2_O, the conformation of the N—H bond in the amide segment is *syn* to the *ortho*-methyl group and *anti* to the *meta*-methyl group in the benzene ring. Further, the conformations of the amide O and the carbonyl O atom of the acid segment are *anti* to the adjacent methyl­ene H atoms. The C=O and O—H bonds of the acid group are *syn* to one another. The structure shows an inter­esting hydrogen-bonding pattern with the water mol­ecule forming hydrogen bonds with three different mol­ecules of the compound. In the crystal, mol­ecules are packed into infinite chains through inter­molecular O—H⋯O and N—H⋯O hydrogen bonds.

## Related literature

For our studies of the effects of substituents on the structures and other aspects of *N*-(ar­yl)-amides, see: Gowda *et al.* (1999 ▶, 2000 ▶, 2010**a* ▶,b* ▶); Saraswathi *et al.* (2011 ▶). For modes of inter­linking carb­oxy­lic acids by hydrogen bonds, see: Leiserowitz (1976 ▶). For packing of mol­ecules involving dimeric hydrogen-bonding associations of each carboxyl group with a centrosymmetrically related neighbor, see: Jagannathan *et al.* (1994 ▶).
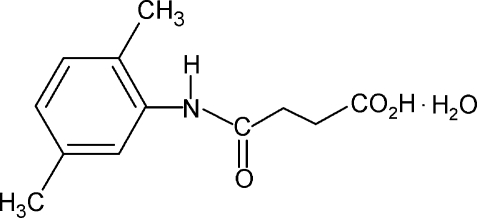


## Experimental

### 

#### Crystal data

C_12_H_15_NO_3_·H_2_O
*M* *_r_* = 239.27
Monoclinic, 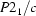

*a* = 22.012 (4) Å
*b* = 6.051 (1) Å
*c* = 9.558 (2) Å
β = 95.90 (1)°
*V* = 1266.3 (4) Å^3^
*Z* = 4
Mo *K*α radiation
μ = 0.09 mm^−1^
*T* = 293 K
0.24 × 0.08 × 0.04 mm

#### Data collection

Oxford Diffraction Xcalibur diffractometer with a Sapphire CCD detector
Absorption correction: multi-scan (*CrysAlis RED*; Oxford Diffraction, 2009 ▶) *T*_min_ = 0.978, *T*_max_ = 0.996
4503 measured reflections
2293 independent reflections
964 reflections with *I* > 2σ(*I*)
*R*_int_ = 0.075

#### Refinement

*R*[*F* ^2^ > 2σ(*F* ^2^)] = 0.099
*wR*(*F* ^2^) = 0.147
*S* = 1.08
2293 reflections
168 parameters
4 restraints
H atoms treated by a mixture of independent and constrained refinement
Δρ_max_ = 0.20 e Å^−3^
Δρ_min_ = −0.22 e Å^−3^

### 

Data collection: *CrysAlis CCD* (Oxford Diffraction, 2009 ▶); cell refinement: *CrysAlis RED* (Oxford Diffraction, 2009 ▶); data reduction: *CrysAlis RED*; program(s) used to solve structure: *SHELXS97* (Sheldrick, 2008 ▶); program(s) used to refine structure: *SHELXL97* (Sheldrick, 2008 ▶); molecular graphics: *PLATON* (Spek, 2009 ▶); software used to prepare material for publication: *SHELXL97*.

## Supplementary Material

Crystal structure: contains datablock(s) I, global. DOI: 10.1107/S1600536811024937/sj5169sup1.cif

Structure factors: contains datablock(s) I. DOI: 10.1107/S1600536811024937/sj5169Isup2.hkl

Supplementary material file. DOI: 10.1107/S1600536811024937/sj5169Isup3.cml

Additional supplementary materials:  crystallographic information; 3D view; checkCIF report

## Figures and Tables

**Table 1:** Hydrogen-bond geometry (Å, °)

*D*—H⋯*A*	*D*—H	H⋯*A*	*D*⋯*A*	*D*—H⋯*A*
N1—H1*N*⋯O1^i^	0.85 (2)	2.10 (2)	2.914 (4)	161 (4)
O2—H2*O*⋯O4^ii^	0.83 (2)	1.81 (2)	2.621 (5)	164 (5)
O4—H41⋯O3	0.84 (2)	2.01 (2)	2.831 (5)	168 (5)
O4—H42⋯O3^iii^	0.83 (2)	2.07 (2)	2.882 (5)	166 (5)

Symmetry codes: (i) 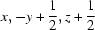; (ii) 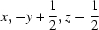; (iii) 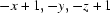.

**Fig. 1.**
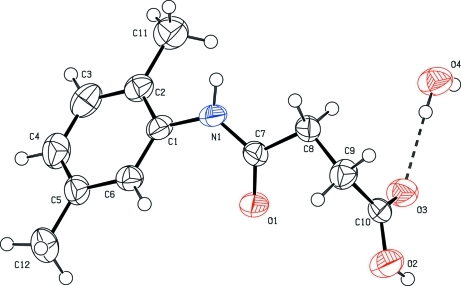
Molecular structure of the title compound, showing the atom labelling scheme and with displacement ellipsoids drawn at the 50% probability level. A hydrogen bond is drawn as a dashed line.

**Fig. 2.**
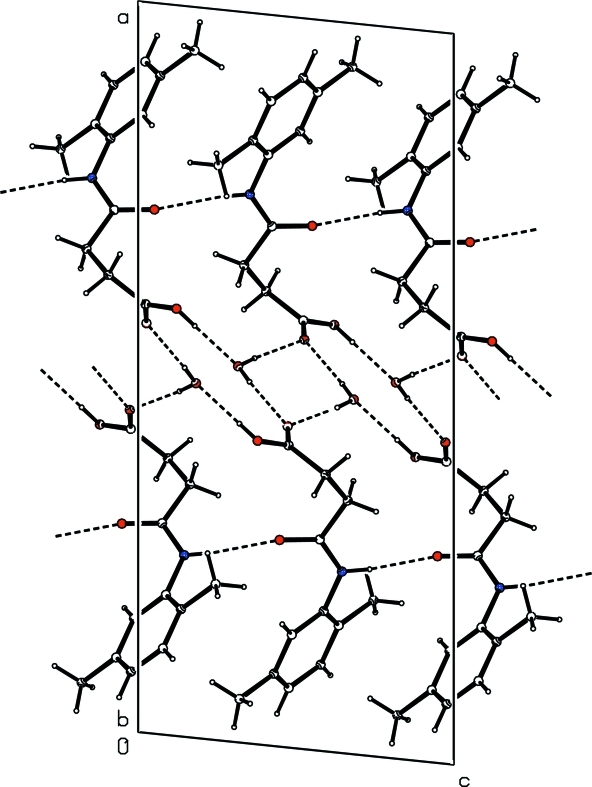
Molecular packing of the title compound with hydrogen bonds shown as dashed lines.

## supplementary crystallographic information

### Comment

The amide and sulfonamide molecules are important constituents of many biologically important compounds. As a part of our studies of the substituent effects on the structures and other aspects of this class of compounds (Gowda *et al.*, 1999, 2000, 2010**a*,b*; Saraswathi *et al.*, 2011), in the present work, the crystal structure of *N*-(2,5-dimethylphenyl)-succinamic acid monohydrate (I) has been determined (Fig. 1). The conformation of the N—H bond in the amide segment is *syn* to the *ortho*–methyl group and *anti* to the *meta*–methyl group in the benzene ring, similar to the *syn* conformation observed between the amide hydrogen and the *ortho*-methyl group in *N*-(2-methylphenyl)succinamic acid (II) (Gowda *et al.*, 2010*b*) and the *anti* conformation observed between the amide hydrogen and the *meta*-methyl group in the benzene ring of *N*-(3-methylphenyl)succinamic acid (III) (Gowda *et al.*, 2010*a*). The conformation of the amide oxygen and the carbonyl oxygen of the acid segment are *anti* to each other. Further, the conformations of these are *anti* to the adjacent methylene H-atoms. The C═O and O—H bonds of the acid group are in *syn* position to each other, similar to that observed in (II) and (III).

The structure shows interesting H–bond pattern with water molecule forming H–bonding with three different molecules of the compound. Intermolecular O—H···O and N—H···O hydrogen bonds pack the molecules into infinite chains in the structure (Table 1, Fig.2). The modes of interlinking carboxylic acids by hydrogen bonds is described elsewhere (Leiserowitz, 1976). The packing of molecules involving dimeric hydrogen bonded association of each carboxyl group with a centrosymmetrically related neighbor has also been observed (Jagannathan *et al.*, 1994).

### Experimental

A solution of succinic anhydride (0.01 mole) in toluene (25 ml) was treated dropwise with the solution of 2,5-dimethylaniline (0.01 mole) also in toluene (20 ml) with constant stirring. The resulting mixture was stirred for about one hour and set aside for an additional hour at room temperature for completion of the reaction. The mixture was then treated with dilute hydrochloric acid to remove the unreacted 2,5-dimethylaniline. The resulting title compound was filtered under suction and washed thoroughly with water to remove the unreacted succinic anhydride and succinic acid. It was recrystallized to constant melting point from ethanol. The purity of the compound was checked and characterized by its infrared and NMR spectra.

Colorless needle like  single crystals used in X-ray diffraction studies were grown in ethanolic solution by slow evaporation at room temperature.

### Refinement

The H atoms of the NH gorup and the water molecule were located in a difference map and their position refined with N—H = 0.86 (2) Å and O—H = 0.82 (2) Å. The other H atoms were positioned with idealized geometry using a riding model with the aromatic C—H = 0.93 Å, methyl C—H = 0.96 Å and methylene C—H = 0.97 Å,,. All H atoms were refined with isotropic displacement parameters (set to 1.2 times of the *U*_eq_ of the parent atom).

### Crystal data

**Table:**

C_12_H_15_NO_3_·H_2_O	*F*(000) = 512
*M**_r_* = 239.27	*D*_x_ = 1.255 Mg m^−^^3^
Monoclinic, *P*2_1_/*c*	Mo *K*α radiation, λ = 0.71073 Å
Hall symbol: -P 2ybc	Cell parameters from 663 reflections
*a* = 22.012 (4) Å	θ = 2.8–27.7°
*b* = 6.051 (1) Å	µ = 0.09 mm^−^^1^
*c* = 9.558 (2) Å	*T* = 293 K
β = 95.90 (1)°	Needle, colourless
*V* = 1266.3 (4) Å^3^	0.24 × 0.08 × 0.04 mm
*Z* = 4	

### Data collection

**Table:**

Oxford Diffraction Xcalibur diffractometer with a Sapphire CCD detector	2293 independent reflections
Radiation source: fine-focus sealed tube	964 reflections with *I* > 2σ(*I*)
graphite	*R*_int_ = 0.075
Rotation method data acquisition using ω scans	θ_max_ = 25.3°, θ_min_ = 2.8°
Absorption correction: multi-scan (*CrysAlis RED*; Oxford Diffraction, 2009)	*h* = −26→26
*T*_min_ = 0.978, *T*_max_ = 0.996	*k* = −7→6
4503 measured reflections	*l* = −11→8

### Refinement

**Table:**

Refinement on *F*^2^	Primary atom site location: structure-invariant direct methods
Least-squares matrix: full	Secondary atom site location: difference Fourier map
*R*[*F*^2^ > 2σ(*F*^2^)] = 0.099	Hydrogen site location: inferred from neighbouring sites
*wR*(*F*^2^) = 0.147	H atoms treated by a mixture of independent and constrained refinement
*S* = 1.08	*w* = 1/[σ^2^(*F*_o_^2^) + (0.0275*P*)^2^ + 1.0954*P*] where *P* = (*F*_o_^2^ + 2*F*_c_^2^)/3
2293 reflections	(Δ/σ)_max_ = 0.004
168 parameters	Δρ_max_ = 0.20 e Å^−^^3^
4 restraints	Δρ_min_ = −0.22 e Å^−^^3^

### Special details

**Table:**

Geometry. All e.s.d.'s (except the e.s.d. in the dihedral angle between two l.s. planes) are estimated using the full covariance matrix. The cell e.s.d.'s are taken into account individually in the estimation of e.s.d.'s in distances, angles and torsion angles; correlations between e.s.d.'s in cell parameters are only used when they are defined by crystal symmetry. An approximate (isotropic) treatment of cell e.s.d.'s is used for estimating e.s.d.'s involving l.s. planes.
Refinement. Refinement of *F*^2^ against ALL reflections. The weighted *R*-factor *wR* and goodness of fit *S* are based on *F*^2^, conventional *R*-factors *R* are based on *F*, with *F* set to zero for negative *F*^2^. The threshold expression of *F*^2^ > σ(*F*^2^) is used only for calculating *R*-factors(gt) *etc*. and is not relevant to the choice of reflections for refinement. *R*-factors based on *F*^2^ are statistically about twice as large as those based on *F*, and *R*- factors based on ALL data will be even larger.

### Fractional atomic coordinates and isotropic or equivalent isotropic displacement parameters (Å^2^)

**Table:**

	*x*	*y*	*z*	*U*_iso_*/*U*_eq_	
C1	0.1917 (2)	0.0833 (9)	0.5880 (4)	0.0380 (13)	
C2	0.1742 (2)	−0.1196 (9)	0.6356 (5)	0.0437 (14)	
C3	0.1180 (3)	−0.2041 (9)	0.5786 (5)	0.0550 (15)	
H3	0.1049	−0.3402	0.6091	0.066*	
C4	0.0817 (2)	−0.0882 (10)	0.4772 (6)	0.0584 (17)	
H4	0.0447	−0.1487	0.4400	0.070*	
C5	0.0990 (2)	0.1137 (10)	0.4304 (5)	0.0464 (15)	
C6	0.1550 (2)	0.2008 (8)	0.4869 (4)	0.0404 (13)	
H6	0.1678	0.3377	0.4568	0.048*	
C7	0.2891 (2)	0.2840 (9)	0.5756 (4)	0.0392 (13)	
C8	0.3462 (2)	0.3619 (9)	0.6640 (4)	0.0439 (14)	
H8A	0.3352	0.4145	0.7538	0.053*	
H8B	0.3738	0.2377	0.6820	0.053*	
C9	0.3788 (2)	0.5434 (8)	0.5942 (5)	0.0474 (14)	
H9A	0.3490	0.6525	0.5578	0.057*	
H9B	0.4069	0.6156	0.6646	0.057*	
C10	0.4137 (2)	0.4669 (10)	0.4765 (5)	0.0387 (13)	
C11	0.2134 (2)	−0.2482 (9)	0.7455 (5)	0.0652 (17)	
H11A	0.2135	−0.1758	0.8349	0.078*	
H11B	0.2544	−0.2560	0.7198	0.078*	
H11C	0.1973	−0.3949	0.7518	0.078*	
C12	0.0596 (2)	0.2444 (10)	0.3213 (5)	0.0719 (19)	
H12A	0.0204	0.1743	0.3034	0.086*	
H12B	0.0789	0.2503	0.2358	0.086*	
H12C	0.0543	0.3918	0.3553	0.086*	
N1	0.24841 (18)	0.1758 (7)	0.6459 (3)	0.0431 (11)	
H1N	0.2554 (19)	0.147 (7)	0.733 (2)	0.052*	
O1	0.28284 (14)	0.3146 (6)	0.4485 (3)	0.0590 (12)	
O2	0.41537 (16)	0.6192 (6)	0.3788 (3)	0.0571 (11)	
H2O	0.436 (2)	0.562 (8)	0.321 (4)	0.068*	
O3	0.43953 (15)	0.2910 (6)	0.4737 (3)	0.0516 (10)	
O4	0.48537 (18)	−0.0138 (6)	0.6831 (4)	0.0569 (11)	
H41	0.472 (2)	0.090 (6)	0.631 (4)	0.068*	
H42	0.505 (2)	−0.111 (6)	0.647 (5)	0.068*	

### Atomic displacement parameters (Å^2^)

**Table:**

	*U*^11^	*U*^22^	*U*^33^	*U*^12^	*U*^13^	*U*^23^
C1	0.042 (4)	0.043 (4)	0.031 (3)	−0.007 (3)	0.008 (2)	−0.001 (3)
C2	0.048 (4)	0.045 (4)	0.040 (3)	0.002 (3)	0.012 (3)	−0.002 (3)
C3	0.060 (4)	0.043 (4)	0.066 (4)	−0.009 (4)	0.023 (3)	−0.003 (3)
C4	0.045 (4)	0.066 (5)	0.064 (4)	−0.014 (4)	0.005 (3)	−0.010 (4)
C5	0.036 (3)	0.062 (5)	0.041 (3)	−0.005 (3)	0.002 (3)	−0.006 (3)
C6	0.042 (3)	0.045 (4)	0.034 (3)	−0.003 (3)	0.004 (2)	0.001 (3)
C7	0.034 (3)	0.052 (4)	0.031 (3)	0.003 (3)	0.001 (2)	0.000 (3)
C8	0.035 (3)	0.067 (4)	0.030 (2)	0.001 (3)	0.002 (2)	−0.003 (3)
C9	0.044 (3)	0.057 (4)	0.041 (3)	−0.005 (3)	0.008 (2)	−0.008 (3)
C10	0.030 (3)	0.054 (4)	0.030 (3)	−0.005 (3)	−0.004 (2)	0.001 (3)
C11	0.068 (4)	0.059 (4)	0.071 (4)	0.011 (4)	0.017 (3)	0.012 (3)
C12	0.042 (4)	0.109 (5)	0.062 (3)	−0.010 (4)	−0.006 (3)	0.003 (4)
N1	0.045 (3)	0.057 (3)	0.028 (2)	−0.011 (3)	0.003 (2)	0.009 (2)
O1	0.048 (2)	0.104 (3)	0.0241 (17)	−0.010 (2)	0.0010 (14)	0.005 (2)
O2	0.066 (3)	0.058 (3)	0.051 (2)	0.009 (2)	0.0200 (18)	0.010 (2)
O3	0.055 (2)	0.056 (3)	0.045 (2)	0.012 (2)	0.0104 (17)	0.005 (2)
O4	0.069 (3)	0.056 (3)	0.048 (2)	0.015 (2)	0.019 (2)	0.0068 (19)

### Geometric parameters (Å, °)

**Table:**

C1—C2	1.378 (6)	C8—H8B	0.9700
C1—C6	1.389 (6)	C9—C10	1.499 (6)
C1—N1	1.427 (6)	C9—H9A	0.9700
C2—C3	1.396 (7)	C9—H9B	0.9700
C2—C11	1.506 (6)	C10—O3	1.208 (6)
C3—C4	1.382 (7)	C10—O2	1.315 (6)
C3—H3	0.9300	C11—H11A	0.9600
C4—C5	1.368 (7)	C11—H11B	0.9600
C4—H4	0.9300	C11—H11C	0.9600
C5—C6	1.397 (6)	C12—H12A	0.9600
C5—C12	1.511 (6)	C12—H12B	0.9600
C6—H6	0.9300	C12—H12C	0.9600
C7—O1	1.223 (4)	N1—H1N	0.852 (18)
C7—N1	1.344 (5)	O2—H2O	0.830 (19)
C7—C8	1.516 (6)	O4—H41	0.836 (19)
C8—C9	1.504 (6)	O4—H42	0.827 (19)
C8—H8A	0.9700		
			
C2—C1—C6	121.6 (5)	C10—C9—C8	114.3 (4)
C2—C1—N1	119.0 (5)	C10—C9—H9A	108.7
C6—C1—N1	119.5 (5)	C8—C9—H9A	108.7
C1—C2—C3	117.6 (5)	C10—C9—H9B	108.7
C1—C2—C11	122.1 (5)	C8—C9—H9B	108.7
C3—C2—C11	120.3 (5)	H9A—C9—H9B	107.6
C4—C3—C2	120.9 (5)	O3—C10—O2	123.6 (5)
C4—C3—H3	119.6	O3—C10—C9	124.5 (5)
C2—C3—H3	119.6	O2—C10—C9	111.8 (5)
C5—C4—C3	121.5 (5)	C2—C11—H11A	109.5
C5—C4—H4	119.3	C2—C11—H11B	109.5
C3—C4—H4	119.3	H11A—C11—H11B	109.5
C4—C5—C6	118.3 (5)	C2—C11—H11C	109.5
C4—C5—C12	122.3 (5)	H11A—C11—H11C	109.5
C6—C5—C12	119.4 (5)	H11B—C11—H11C	109.5
C1—C6—C5	120.1 (5)	C5—C12—H12A	109.5
C1—C6—H6	119.9	C5—C12—H12B	109.5
C5—C6—H6	119.9	H12A—C12—H12B	109.5
O1—C7—N1	123.9 (4)	C5—C12—H12C	109.5
O1—C7—C8	120.6 (4)	H12A—C12—H12C	109.5
N1—C7—C8	115.5 (4)	H12B—C12—H12C	109.5
C9—C8—C7	112.7 (4)	C7—N1—C1	126.8 (4)
C9—C8—H8A	109.1	C7—N1—H1N	122 (3)
C7—C8—H8A	109.1	C1—N1—H1N	111 (3)
C9—C8—H8B	109.1	C10—O2—H2O	104 (4)
C7—C8—H8B	109.1	H41—O4—H42	117 (5)
H8A—C8—H8B	107.8		
			
C6—C1—C2—C3	0.1 (7)	C4—C5—C6—C1	0.0 (7)
N1—C1—C2—C3	179.0 (4)	C12—C5—C6—C1	179.6 (4)
C6—C1—C2—C11	−179.8 (4)	O1—C7—C8—C9	21.9 (7)
N1—C1—C2—C11	−0.9 (7)	N1—C7—C8—C9	−160.2 (4)
C1—C2—C3—C4	0.4 (7)	C7—C8—C9—C10	−75.3 (5)
C11—C2—C3—C4	−179.7 (4)	C8—C9—C10—O3	−34.4 (7)
C2—C3—C4—C5	−0.7 (8)	C8—C9—C10—O2	148.7 (4)
C3—C4—C5—C6	0.5 (7)	O1—C7—N1—C1	−1.3 (8)
C3—C4—C5—C12	−179.1 (5)	C8—C7—N1—C1	−179.1 (5)
C2—C1—C6—C5	−0.3 (7)	C2—C1—N1—C7	139.5 (5)
N1—C1—C6—C5	−179.2 (4)	C6—C1—N1—C7	−41.6 (7)

### Hydrogen-bond geometry (Å, °)

**Table:**

*D*—H···*A*	*D*—H	H···*A*	*D*···*A*	*D*—H···*A*
N1—H1N···O1^i^	0.85 (2)	2.10 (2)	2.914 (4)	161 (4)
O2—H2O···O4^ii^	0.83 (2)	1.81 (2)	2.621 (5)	164 (5)
O4—H41···O3	0.84 (2)	2.01 (2)	2.831 (5)	168 (5)
O4—H42···O3^iii^	0.83 (2)	2.07 (2)	2.882 (5)	166 (5)

Symmetry codes: (i) *x*, −*y*+1/2, *z*+1/2; (ii) *x*, −*y*+1/2, *z*−1/2; (iii) −*x*+1, −*y*, −*z*+1.
